# A novel risk signature with 6 RNA binding proteins for prognosis prediction in patients with glioblastoma

**DOI:** 10.1097/MD.0000000000028065

**Published:** 2021-12-03

**Authors:** Qian-Rong Huang, Jian-Wen Li, Xin-Bin Pan

**Affiliations:** a Department of Neurosurgery, Guangxi Medical University Cancer Hospital, Nanning, Guangxi, P.R. China; b Department of Radiation Oncology, Guangxi Medical University Cancer Hospital, Nanning, Guangxi, P.R. China.

**Keywords:** glioblastoma, prognosis, RNA binding protein, signature

## Abstract

Recent studies suggested that RNA binding proteins (RBPs) were related to the tumorigenesis and progression of glioma. This study was conducted to identify prognostic RBPs of glioblastoma (GBM) and construct an RBP signature to predict the prognosis of GBM.

Univariate Cox regression analysis was carried out to identify the RBPs associated with overall survival of GBM in the The Cancer Genome Atlas (TCGA), GSE16011, and Repository for Molecular Brain Neoplasia data (Rembrandt) datasets, respectively. Overlapping RBPs from the TCGA, GSE16011, and Rembrandt datasets were selected. The biological role of prognostic RBPs was assessed by Gene Ontology, Kyoto Encyclopedia of Genes and Genomes, and protein–protein interaction analyses. Least absolute shrinkage and selection operator regression analysis and multivariate Cox regression analysis were used to construct an RBP-related risk signature. The prognostic value of RBP signature was measured by Kaplan–Meier method and time-dependent receiver operating characteristic curve. A nomogram based on independent prognostic factors was established to predict survival for GBM. The CGGA cohort was used as the validation cohort for external validation.

This study identified 27 RBPs associated with the prognosis of GBM and constructed a 6-RPBs signature. Kaplan–Meier curves suggested that high-risk score was associated with a poor prognosis. Area under the curve of 1-, 3-, and 5-year overall survival was 0.618, 0.728, and 0.833 for TCGA cohort, 0.655, 0.909, and 0.911 for GSE16011 cohort, and 0.665, 0.792, and 0.781 for Rembrandt cohort, respectively. A nomogram with 4 parameters (age, chemotherapy, O^6^-methylguanine-DNA methyltransferase promoter status, and risk score) was constructed. The calibration curve showed that the nomogram prediction was in good agreement with the actual observation.

The 6-RBPs signature could effectively predict the prognosis of GBM, and our findings supplemented the prognostic index of GBM to a certain extent.

## 1 Introduction

Glioblastoma (GBM) is a glioma subtype with the highest degree of malignancy and the worst prognosis, with a 5-year survival rate of 6.8%.^[1]^ Although the standard treatment regimen is available, including surgery, postoperative radiotherapy, and chemotherapy, the median survival of GBM patients is still less than 2 years.^[2]^ Currently, there is no effective treatment for GBM due to its high aggressiveness, high heterogeneity, and easy tolerance to treatment.^[3–5]^ Hence, it is urgent to find novel prognostic markers and therapeutic targets for this catastrophic tumor.

RNA binding proteins (RBPs) are a class of proteins characterized by interaction with target RNAs, of which about 40% of RBP genes are widely expressed in vivo, and the remaining RBP genes are tissue-specific.^[6]^ Up to now, more than 1500 RBP genes have been found in the human genome, which plays an important role in regulating the expression of target genes at the post-transcriptional level and maintaining intracellular homeostasis.^[7,8]^ In recent years, a large number of evidence suggest that RBPs are involved in the genesis and development of cancer.^[9]^ Moreover, increasing studies have also revealed a link between RBPs and glioma progression.^[10,11]^ Several recent studies have systematically evaluated the expression patterns and clinical prognostic value of RBPs in gliomas, and some promising RBP-related signatures have been identified.^[12–14]^ As such, targeting RBPs may be a promising treatment strategy for glioma in the future. However, few studies have systematically analyzed the prognostic role of RBPs in GBM.

In the present study, we first screened out the RBPs associated with GBM prognosis through The Cancer Genome Atlas (TCGA), GSE16011, and Repository for Molecular Brain Neoplasia data (Rembrandt) datasets. Then, Gene Ontology (GO), Kyoto gene and genome encyclopedia (KEGG), and protein–protein interaction (PPI) analyses were used to investigate the potential biological role of these prognostic RBPs in GBM. Then, we developed a prognostic risk signature in the TCGA dataset as the training cohort and validated it using the GSE16011 and Rembrandt dataset as the validation cohort. To further investigate the clinical application value of the RPB signature, we constructed a nomogram to predict the 1-, 3-, and 5-year survival rates of patients with GBM.

## 2 Materials and methods

### 2.1 Data collection

The datasets used in this study were all available to the public. The normalized mRNA expression data and clinical data of GBM patients in the TCGA (GBM dataset, HG-UG133A), GSE16011, and Rembrandt dataset were obtained from the Gliovis online database (http://gliovis.bioinfo.cnio.es/).^[15]^ The glioma dataset (mRNAseq 693) in the Chinese Glioma Genome Atlas (CGGA) database was used as the nomogram validation cohort, and relevant expression data and clinical information were downloaded from the CGGA website (http://www.cgga.org.cn/index.jsp). We obtained a list of 1542 RBPs from a previous public study.^[7]^

### 2.2 Identification of prognostic-related RBPs in GBM

To reduce the interference of other factors in the survival analysis, only patients with postoperative overall survival [OS]) ≥90 days were included in this study. Univariate Cox regression analysis was used to select prognostic-related RBPs in the TCGA, GSE16011, and Rembrandt datasets, respectively, with *P* value <.05 being the threshold of significance. To ensure accuracy, overlapping RBPs in these 3 datasets were extracted for subsequent analysis and visualized using a Venn diagram.

### 2.3 Enrichment analysis of prognostic-related RBPs

The GO function and KEGG pathway of the prognostic-related RBPs were analyzed by R clusterProfiler package, and the results with *P* value <.05 were considered statistically significant. We then used the STRING database (https://string-db.org/) to explore the interactions between these prognostic RBPs proteins and the PPI network was visualized using Cytoscape (version 3.8.0).

### 2.4 Prognostic RBP signature construction and assessment

The prognostic-related RBPs were included for least absolute shrinkage and selection operator (LASSO) regression analysis to screen out the optimal gene combination in the training cohort (TCGA). The prognostic RBP signature of GBM were constructed using multivariate Cox proportional risk regression analysis via step function in R. The risk score for each GBM patient was calculated by the following formula: risk score = (expression_mRNA1_ ∗ Coef_mRNA1_)  +  (expression_mRNA2_ ∗ Coef_mRNA2_)  + … + (expression_mRNAn_ ∗ Coef_mRNAn_).^[16]^ Then, the patients were divided into high-risk and low-risk groups according to the median value of the risk score as a cutoff. Kaplan–Meier method was used to measure the difference of OS between the high-risk and the low-risk groups, and the prediction accuracy of risk score was evaluated using the time-dependent receiver operating characteristic (ROC) curve, which was verified in the validation cohorts (GSE16011 and Rembrandt datasets). In addition, the association between risk score and clinical characteristics was investigated through the TCGA cohort.

### 2.5 Independent prognostic value of the RBP signature

Multivariate Cox regression analyses were performed to assess the independent prognostic value of the RBP-related risk score in TCGA, GSE16011, and Rembrandt cohorts, respectively. To further investigate the clinical application value of the risk score, a prognostic nomogram was constructed based on the independent prognostic factors of the TCGA cohort to predict the 1-, 3-, and 5-years survival of GBM patients. The CGGA cohort was used for external validation. The accuracy of the nomogram was evaluated using calibration curves.

### 2.6 Statistical analysis

The statistical analysis and graphs in this study were performed using R (version 3.6.3). Quantitative data were expressed as mean ± SD. The Wilcoxon test was used to measure the differences between 2 groups, and the Kruskal–Wallis test was used for multigroup comparisons. Kaplan–Meier curve and Log–Rank test was used to measure the difference of OS between the high-risk and the low-risk groups. Univariate and multivariate Cox regression analyses were performed to identify the independent prognostic factors in GBM patients. The RMS R package was used for the construction and evaluation of the nomogram. *P* value <.05 was considered statistically significant.

Ethical review and approval were waived for this study, due to all data deriving from public databases.

## 3 Result

### 3.1 Identification and enrichment analysis of prognostic-related RBPs

**Figure 1:**
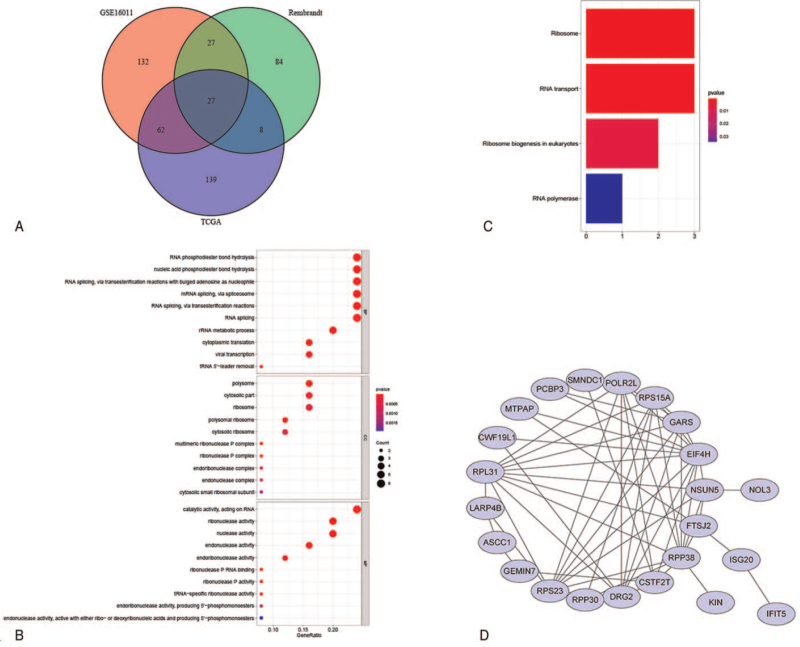
Identification and enrichment analysis of prognostic-related RBPs. (A) 27 overlapping prognostic RBPs were identified from the TCGA, GSE16011, and Rembrandt datasets. (B) The top 10 significantly enriched GO annotations associated with these RBPs. (C) KEGG analysis of 27 prognostic RPBs. (D)The PPI network visualizes their interactions. GO = Gene Ontology, KEGG = Kyoto Encyclopedia of Genes and Genomes, PPI = protein–protein interaction, RBP = RNA binding protein, Rembrandt = Repository for Molecular Brain Neoplasia data, TCGA = The Cancer Genome Atlas.

We obtained 236, 248, and 146 prognostic RBPs from the TCGA, GSE16011, and Rembrandt datasets, respectively, and found a total of 27 RBPs overlapping (Fig. 1A). The potential functions and pathways of these RBPs in patients with GBM were evaluated using GO and KEGG analyses. GO analysis indicated that these genes were enriched in RNA phosphodiester bond hydrolysis, nucleic acid phosphodiester bond hydrolysis, RNA splicing, rRNA metabolic process, cytoplasmic translation, ribonuclease P complex, and ribonuclease P RNA binding (Fig. 1B). In addition, the 27 RBPs were primarily enriched in the ribosome, RNA transport, ribosome biogenesis in eukaryotes, and RNA polymerase KEGG pathways (Fig. 1C). Then, a PPI network was constructed via the String database, and the interaction among these RBPs was shown in Fig. 1D.

### 3.2 Construction and validation of a prognostic RPB signature in GBM

**Figure 2:**
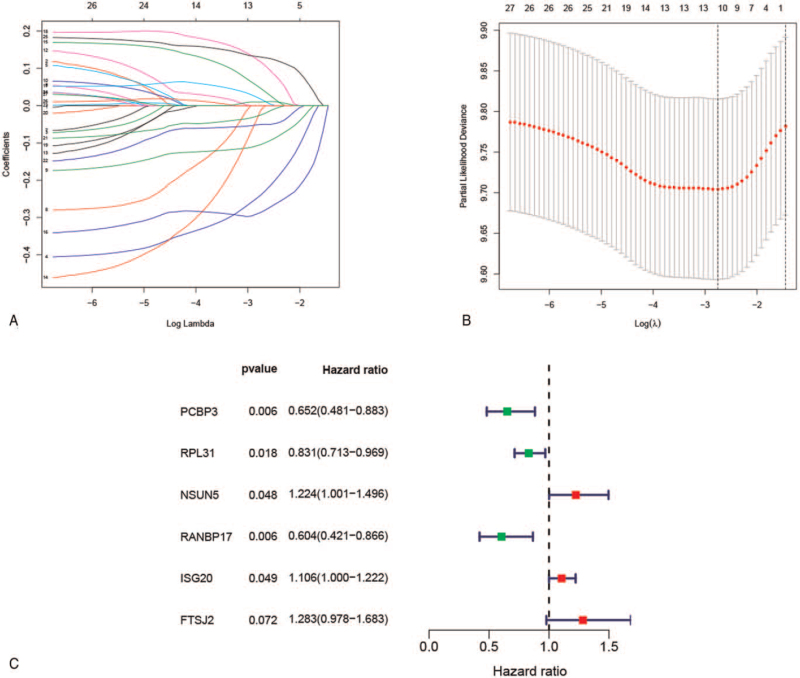
Construction of prognostic RPB signature in the TCGA dataset. (A) The LASSO model showed log (lambda) values for 27 prognostic RBPs. (B) Optimal parameter selection of GBM in Lasso model. (C) A 6-RBPs signature was constructed using multivariate regression analysis. GBM = glioblastoma, LASSO = least absolute shrinkage and selection operator, RBP = RNA binding protein, TCGA = The Cancer Genome Atlas.

**Figure 3:**
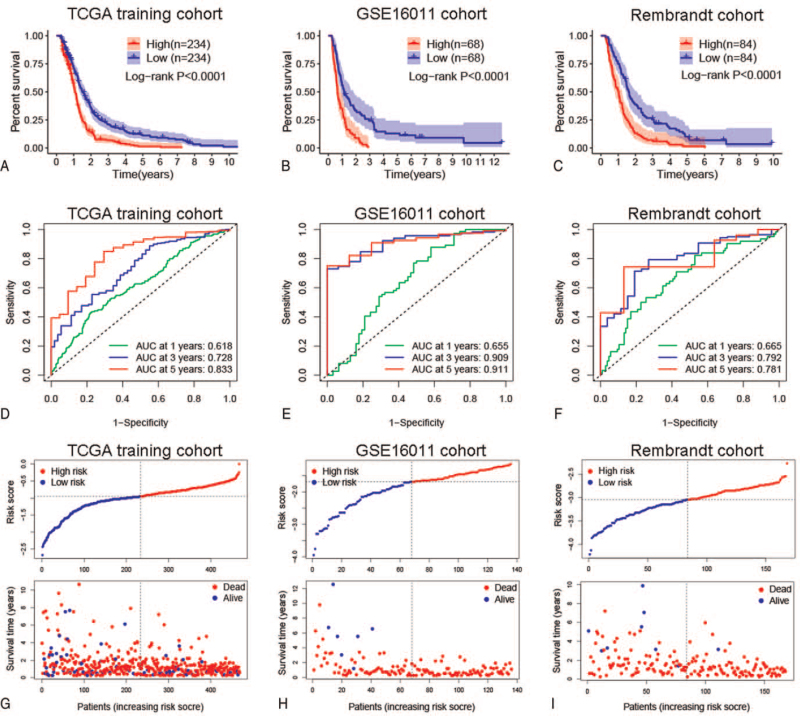
The relationship between RBP signature and prognosis of GBM patients. (A–C) Kaplan–Meier curves showed that GBM patients with high-risk score had a worse prognosis in all cohorts. (D–F) The ROC curve evaluated the accuracy of the prediction of the risk score. (G–I) The risk score and survival status distributions of GBM patients in each cohort. GBM = glioblastoma, RBP = RNA binding protein, ROC = receiver operating characteristic.

A total of 468 GBM patients were enrolled in the TCGA training cohort. We used LASSO regression analysis to analyze the 27 prognostic RBPs, and then 11 of them were selected (Fig. 2A–B). Finally, a 6-RBPs signature was established by multivariate Cox regression analysis (Fig. 2C). The regression coefficients of these 6 RBPs are shown in Table 1. The risk score for each GBM patient was calculated as follows: risk score = (−0.4284 ∗ ExpressionPCBP3)  + (−0.1850 ∗ ExpressionRPL31)  + (0.2021 ∗ ExpressionNSUN5)  + (−0.5048 ∗ ExpressionRANBP17)  + (0.1006 ∗ ExpressionISG20)  + (0.2493 ∗ ExpressionFTSJ2). The patients were divided into high-risk and low-risk groups according to the median value of the risk score. The Kaplan–Meier curve suggested that GBM patients in the high-risk group had a worse prognosis than those in the low-risk group (*P* < .0001) (Fig. 3A). Moreover, the ROC curves showed that the area under the curve for risk score to predict 1-, 3-, and 5-year survival was 0.618, 0.728, and 0.833, respectively (Fig. 3D). We then further evaluated the prediction performance of the risk score in 2 independent external validation cohorts, including the GSE16011 cohort (n = 136) and Rembrandt cohort (n = 168). Consistent with the above results, the OS of GBM patients in the high-risk group was significantly shorter than that of patients in the low-risk group in both 2 validation cohorts (Fig. 3B-C). The area under the curve for 1-, 3-, and 5-year OS was 0.655, 0.909, and 0.911 in the GSE16011 cohort (Fig. 3E) and 0.665, 0.792, and 0.781 in the Rembrandt cohort (Fig. 3F), respectively. The risk score and survival status distributions of GBM patients in each cohort were shown in Fig. 3G–I. Taken together, these results suggested that the 6-RBPs signature could effectively predict clinical outcomes in patients with GBM.

**Table 1:** Genes contained in the RBP signature.

Gene symbol	Full name	Coef	HR	95% CI	*P* value
PCBP3	Poly (rC) Binding Protein 3	−0.4284	0.6515	0.4809–0.8828	.0057
RPL31	Ribosomal Protein L31	−0.185	0.8311	0.7128–0.9691	.0182
NSUN5	NOP2/Sun RNA Methyltransferase 5	0.2021	1.2240	1.0014–1.4962	.0484
RANBP17	RAN Binding Protein 17	−0.5048	0.6036	0.4207–0.8661	.0061
ISG20	Interferon Stimulated Exonuclease Gene 20	0.1006	1.1058	1.0004–1.2223	.0492
FTSJ2	FtsJ RNA Methyltransferase Homolog 2	0.2493	1.2831	0.9780–1.6834	.0720

### 3.3 The relationship between RPB signature and GBM clinical characteristics

**Figure 4:**
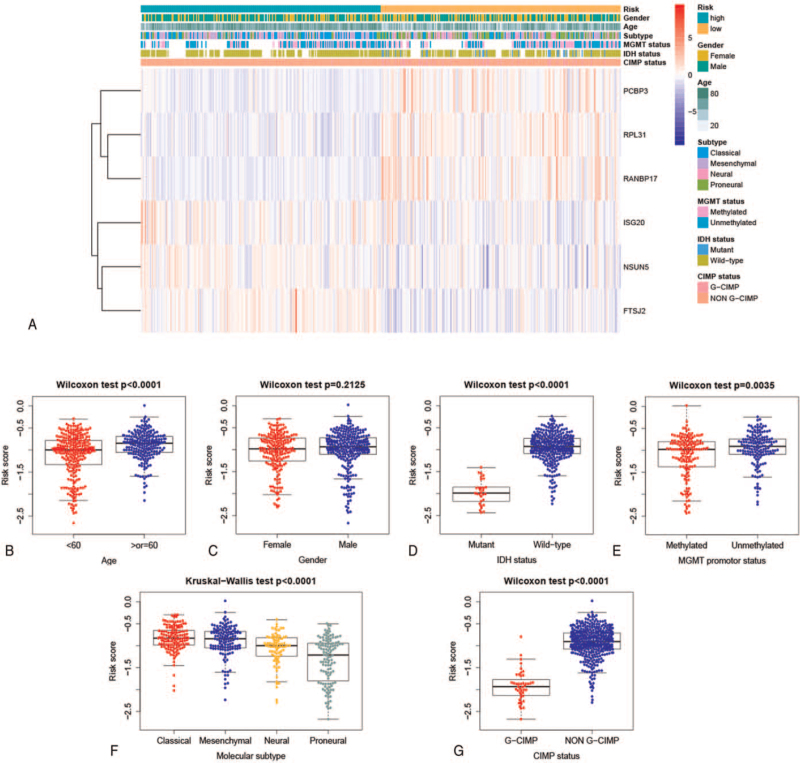
The RBP signature was correlated with clinicopathological characteristics of GBM. (A) The heat map showed the distribution of clinical features, risk score and the 6 selected RBPs levels in the TCGA training cohort. Significant differences were found in risk score among GBM patients categorized by (B) age, (D) IDH status, (E) MGMT promoter status, (F) molecular subtype, and (G) CIMP status, and no differences between (C) genders. GBM = glioblastoma, CIMP = CpG island methylator phenotype, IDH = isocitrate dehydrogenase, MGMT = O^6^-methylguanine-DNA methyltransferase, RBP = RNA binding protein, TCGA = The Cancer Genome Atlas.

To analyze the relationship between RBP signature and clinical characteristics of GBM, a heat map was used to visualize the distribution of the risk score, common clinical features, and 6 selected RBPs levels in the TCGA training cohort (Fig. 4A). The results showed that in the high-risk group, the expression levels of ISG20, NSUN5, and FTSJ2 were higher, while the expression levels of PCBP3, RPL31, and RANBP17 were lower. We also found a higher proportion of patients with isocitrate dehydrogenase (IDH) wild-type, mesenchymal subtype, NON Glioma CpG island methylator phenotype (G-CIMP), and older age in the high-risk group, which are generally considered to be subtypes with worse prognosis.^[4]^ Further, we compared the risk score between different subgroups in the TCGA training cohort. The result showed that there were significant differences in the risk score among GBM patients categorized by age, IDH status, O^6^-methylguanine-DNA methyltransferase (MGMT) promoter status, molecular subtype, and CIMP status, but no differences between genders (Fig. 4B–G), suggesting a higher level of risk score in subtypes with poorer prognosis of GBM.

### 3.4 RPB signature as an independent prognostic factor for GBM patients

Univariate and multivariate Cox regression analyses were used to evaluate the independent prognostic value of the RPB signature and clinical characteristics. A total of 259 patients with complete clinical information were enrolled in the TCGA cohort. The results of the univariate analysis showed that age, CIMP status, IDH status, MGMT promoter status, molecular subtype, chemotherapy, and risk score was closely related to OS of GBM patients (*P* < .05). Subsequent multivariate analysis showed that the risk score was an independent predictor of GBM patient's OS (HR = 1.684, 95% CI = 1.243–2.283, *P* < .001) (Table 2). Furthermore, consistent results were observed in the GSE16011 cohort (HR = 1.587, 95% CI = 1.048–2.404, *P* = .029) (Table 3) and the Rembrandt cohort (HR = 1.880, 95% CI = 1.305–2.706, *P* < .001) (Table 4). These results confirmed the independent clinical prognostic significance of the RBP signature in GBM.

**Table 2:** Univariate and multivariate Cox regression analyses were used to identify independent prognostic factors in the TCGA cohort.

Covariates	Univariate analysis		Multivariate analysis	
	HR (95% CI)	*P*	HR (95% CI)	*P*
Gender	1.012 (0.764–1.342)	.931		
Age	1.034 (1.023–1.045)	<.001	1.023 (1.011–1.036)	<.001
CIMP status	0.304 (0.177–0.521)	<.001	0.232 (0.031–1.737)	.155
IDH status	0.340 (0.196–0.590)	<.001	2.460 (0.316–19.141)	.39
MGMT promotor status	0.667 (0.504–0.881)	.004	0.726 (0.547–0.965)	.028
Molecular subtype	0.866 (0.770–0.975)	.018	1.082 (0.947–1.236)	.248
Radiotherapy	0.569 (0.317–1.022)	.059		
Chemotherapy	0.567 (0.392–0.819)	.003	0.616 (0.418–0.906)	.014
Risk score	1.954 (1.464–2.608)	<.001	1.684 (1.243–2.283)	<.001

**Table 3:** Univariate and multivariate Cox regression analyses were used to identify independent prognostic factors in the GSE16011 cohort.

Covariates	Univariate analysis		Multivariate analysis	
	HR (95% CI)	*P*	HR (95% CI)	*P*
Gender	1.051 (0.728–1.517)	.79		
Age	1.039 (1.024–1.055)	<.001	1.029 (1.013–1.046)	<.001
CIMP status	0.308 (0.180–0.528)	<.001	0.425 (0.218–0.830)	.012
IDH status	0.747 (0.584–0.956)	.02	0.846 (0.673–1.064)	.153
Molecular subtype	0.761 (0.660–0.878)	<.001	1.059 (0.878–1.278)	.547
Risk score	2.446 (1.679–3.564)	<.001	1.587 (1.048–2.404)	.029

**Table 4:** Univariate and multivariate Cox regression analyses were used to identify independent prognostic factors in Rembrandt cohort.

Covariates	Univariate analysis		Multivariate analysis	
	HR (95% CI)	*P*	HR (95% CI)	*P*
CIMP status	0.365 (0.149–0.892)	.027	0.521 (0.204–1.331)	.173
Molecular subtype	0.810 (0.707–0.928)	.002	0.955 (0.813–1.121)	.574
Risk score	2.105 (1.527–2.900)	<.001	1.880 (1.305–2.706)	<.001

### 3.5 Construction and evaluation of a nomogram to predict the prognosis of GBM

**Figure 5:**
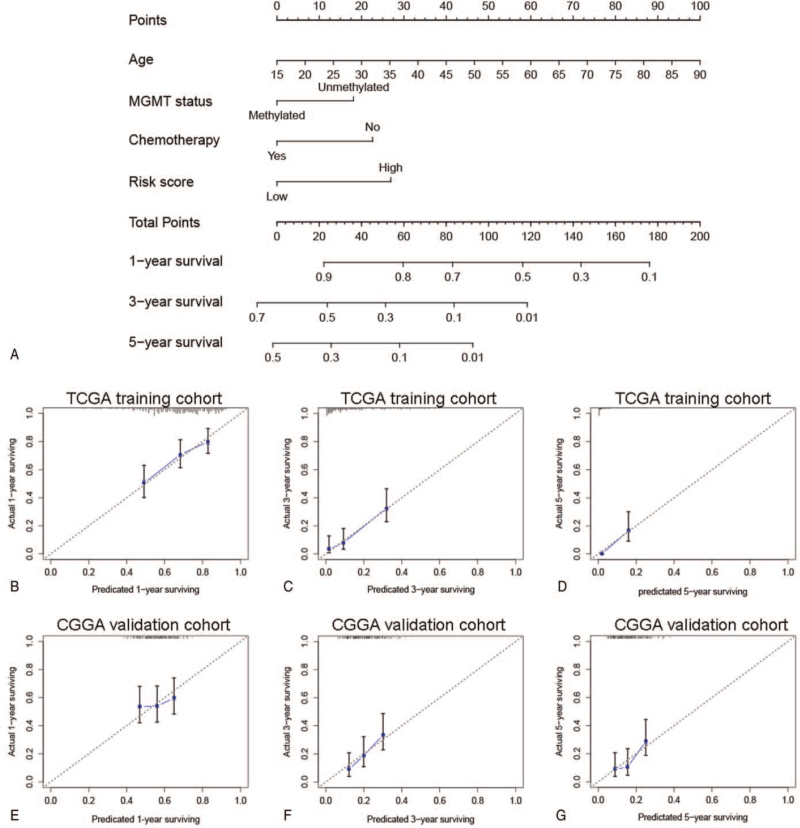
Construction and validation of a nomogram. (A) A normogram was constructed for predicting 1-, 3 -, and 5-years survival in the TCGA training cohort. (B–D) The calibration plot for internal assessment. (E–G) The calibration plot for external validation. TCGA = The Cancer Genome Atlas.

To further explore the clinical application value of the RBP signature, we next constructed a nomogram based on the independent prognostic factors (age, chemotherapy, MGMT promoter status, and risk score) of GBM in the TCGA training cohort (n = 259) (Fig. 5A). A total of 175 patients in the CGGA dataset were included for external validation. Then, the calibration curve was used to evaluate the performance of the nomogram. As shown in Fig. 5B–G, the predictions for 1-, 3-, and 5-years OS of the nomogram had a high consistency with the actual observations, both in the TCGA training cohort and CGGA testing cohort. It was indicated that the nomogram had a good performance in predicting GBM prognosis.

## 4 Discussion

Although there have been some advances in the treatment of GBM over the past few decades, survival did not improve significantly.^[17]^ Recent studies have shown that RBPs are closely related to the occurrence and development of GBM. Correa et al^[18]^ revealed that SNRPB was involved in RNA processing, DNA repair, and chromatin remodeling. SNRPB was an oncogenic GBM candidate. Velasco et al^[19]^ reported that when the normal interaction between RBP MSI1 and miR-137 was unbalanced, it could lead to different outcomes in GBM cells, including self-renewal, proliferation, and tumorigenesis.^[19]^ The RBP SERBP1 expression is up-regulated in GBM tissue, and GBM patients with high SERBP1 expression have a worse prognosis and reduced responsiveness to radiotherapy and chemotherapy.^[20]^ Downregulation of RBP SNRPG induces GBM cell cycle arrest and increases sensitivity to chemotherapy.^[21]^ GBM patients with high expression levels of SRSF3 have a poor prognosis, and SRSF3 is a key regulator of glioma-associated selective splicing.^[22]^ To date, the prognostic role of most RBPs in GBM remains unclear. Therefore, a systematic analysis of RBPs may have important clinical significance for this refractory tumor.

In the current study, we identified 27 RBPs associated with GBM prognosis in multiple datasets, and these key RPBs may be closely associated with GBM progression. Functional analysis suggested that these key RBPs in GBM may be involved in interactions with target RNA, including RNA splicing, translation, and metabolic processes, thereby regulating gene expression at the post-transcriptional level. The subsequent PPI network showed correlations among these prognostic RBPs. We then constructed a prognostic signature with 6 RBPs for GBM patients, and our analysis found that the OS of patients in the high-risk group was significantly shorter than that of patients in the low-risk group, and more importantly, consistent results were obtained from both validation groups. Time-dependent ROC curves showed that the RPB signature had high predictive accuracy and could easily distinguish GBM patients with good prognosis from those with poor prognosis. We also observed a correlation between risk score and clinicopathologic features in GBM patients, including age, IDH1 status, CIMP status, molecular subtype, and MGMT promoter status. Furthermore, multivariate Cox regression analysis suggested that the RBP signature was an independent risk factor for GBM survival. These results highlight the potential of this RBP signature as a new prognostic marker in GBM patients. So far, the RBP signature has been constructed for a variety of cancers, such as glioma,^[12–14]^ liver cancer,^[23]^ lung cancer,^[24]^ and bladder cancer.^[25]^ However, for GBM, the subtype of glioma with the worst prognosis, there are no relevant studies to our knowledge, so the current study is a useful addition.

In this study, the RBP-related prognostic signature we constructed, including PCBP3, RPL31, NSUN5, RANBP17, ISG20, and FTSJ2. Previous studies have suggested a link between these genes and tumors. Ger et al^[26]^ reported that pancreatic cancer with high PCBP3 expression had a better prognosis and that PCBP3 expression was correlated with tumor stage. A previous study showed that RPL31 was overexpressed in prostate cancer tissues, and silencing RPL31 inhibited tumor cell growth and cycle progression, which was due to its regulation of the P53 pathway.^[27]^ NSUN5 is an RNA methylation transferase, which acts as a tumor suppressor in glioma, but NSUN5 expression is down-regulated due to CpG island hypermethylation of the promoter. Interestingly, patients with low NSUN5 expression had better clinical outcomes in all grades of gliomas, possibly because this epigenetic silencing increased the sensitivity of glioma to specific treatments.^[28,29]^ In our study, NSUN5 expression was also shown to be a prognostic risk factor for GBM. Consistent with our data, 2 previous studies have suggested that high expression of RANBP17 is associated with a favorable prognosis for glioma.^[12,30]^ Gao et al^[31]^ found that ISG20 expression increased with the increase of glioma malignancy, and high ISG20 expression predicted a poor prognosis. Moreover, ISG20 improved the immune capacity in the tumor environment by promoting tumor-induced immune response and immune cell infiltration.^[31]^ Another study also suggested that ISG20 expression was inversely associated with survival in glioma patients.^[14]^ In this study, our results were consistent with these conclusions. For the RBP FTSJ2, previous studies had shown that it had heat shock protein properties and was considered as a tumor suppressor gene in lung cancer.^[32,33]^ So far, studies on these 6 important RBPs in GBM are limited, and further exploration is necessary.

The nomogram model is a novel risk quantification tool that is widely used in oncology,^[34]^ and it should be convenient and accurate.^[35]^ In our study, 4 parameters were included in the RBP-related nomogram, 3 of which were commonly used clinicopathological features (age, chemotherapy, and MGMT promoter status), indicating that our nomogram was concise. The calibration curve confirmed that the nomogram prediction was consistent with the actual observation, and more importantly, the external verification result was consistent, indicating that the RBP-related nomogram could accurately assess the prognosis of GBM. However, the limitation of this study should be considered. Firstly, due to the limitations of these public databases, some important clinical parameters, such as the extent of the tumor resection, tumor size and location, and preoperative status, are not provided, which may affect the construction and efficacy of the nomogram. Secondly, this is a retrospective study, and the role of the RBPs signature and the nomogram needs to be confirmed by basic experiments and a large clinical cohort.

## 5 Conclusion

In summary, our study evaluated the prognostic values of RBPs in GBM and constructed a 6-RBPs signature for predicting the GBM patients’ prognosis. The RBP signature was closely related to the clinicopathological features and was an independent prognostic factor. Our data complement the prognostic indicators of GBM. The results may provide a novel idea for individualized treatment of this refractory tumor.

## Author contributions

**Conceptualization**: Qian-Rong Huang, Xin-Bin Pan.

**Data curation**: Qian-Rong Huang, Jian-Wen Li.

**Formal analysis**: Qian-Rong Huang.

**Methodology**: Qian-Rong Huang, Jian-Wen Li.

**Resources**: Jian-Wen Li.

**Software**: Qian-Rong Huang, Jian-Wen Li.

**Visualization**: Qian-Rong Huang, Xin-Bin Pan.

Writing – original draft: Qian-Rong Huang.

Writing – review & editing: Xin-Bin Pan.

**Conceptualization:** Qian-Rong Huang, Xin-Bin Pan.

**Data curation:** Qian-Rong Huang, Jian-Wen Li.

**Formal analysis:** Qian-Rong Huang.

**Funding acquisition:** Qian-Rong Huang.

**Investigation:** Qian-Rong Huang.

**Resources:** Jian-Wen Li.

**Software:** Jian-Wen Li.

**Supervision:** Jian-Wen Li.

**Validation:** Jian-Wen Li.

**Writing – original draft:** Qian-Rong Huang.

**Writing – review & editing:** Xin-Bin Pan.
